# Genetic Insights and Clinical Implications of *NEU1* Mutations in Sialidosis

**DOI:** 10.3390/genes16020151

**Published:** 2025-01-25

**Authors:** Mei-Ling Peng, Siu-Fung Chau, Jia-Ying Chien, Peng-Yeong Woon, Yu-Chen Chen, Wai-Man Cheang, Hsien-Yang Tsai, Shun-Ping Huang

**Affiliations:** 1 Department of Ophthalmology, Taichung Tzu Chi Hospital, Taichung 427213, Taiwan; pml02046@gmail.com (M.-L.P.); cipechau@gmail.com (S.-F.C.); moonimi0219@yahoo.com.tw (Y.-C.C.); adycheang@gmail.com (W.-M.C.); choihinyeung@tzuchi.com.tw (H.-Y.T.); 2 Department of Biochemical Science and Technology, National Chiayi University, Chiayi 600355, Taiwan; jychien416@gmail.com; 3 Department of Molecular Biology and Human Genetics, Tzu Chi University, Hualien 970374, Taiwan; woon07@gms.tcu.edu.tw

**Keywords:** sialidosis, NEU1, lysosomal storage disorder, genetic heterogeneity, enzyme replacement therapy, gene therapy

## Abstract

Sialidosis is a rare autosomal recessive lysosomal storage disorder caused by mutations in the *NEU1* gene, resulting in deficient neuraminidase-1 activity and the subsequent accumulation of sialylated compounds in lysosomes. This review comprehensively analyzes the genetic and clinical heterogeneity associated with sialidosis, emphasizing the distinction between the milder type I form and the more severe type II form. Over 90 pathogenic *NEU1* variants, predominantly missense mutations, have been identified, highlighting significant phenotypic diversity. Advancements in genomic sequencing technologies have facilitated the identification of known and novel mutations, with population-specific insights elucidating ethnic variability in symptomatology and genetic profiles. Recent case studies, including a novel compound heterozygous variant, further illustrate the complexity of the genotype–phenotype correlations. Emerging therapeutic approaches, such as enzyme replacement therapy and adeno-associated virus-mediated gene therapy, demonstrate promising potential for restoring neuraminidase-1 function and improving outcomes in preclinical models. This review emphasizes the critical role of genetic analysis in diagnosis and management while advocating for continued research into the molecular mechanisms underlying sialidosis to enable the development of targeted, personalized treatments.

## 1. Introduction

### 1.1. Background on Sialidosis and NEU1 Gene

Neuraminidase 1 (NEU1) is an essential enzyme expressed by the *NEU1* gene and has a critical functional role in lysosomal activity. This enzyme catalyzes the breakdown of sialic acid-containing substances within lysosomes by hydrolyzing terminal sialic acid residues from glycoproteins and glycolipids. A reduction in the enzymatic function of NEU1 results in the accumulation of sialic acid in various tissues, which is associated with several clinical manifestations indicative of lysosomal storage diseases [1,2].

Sialidosis, classified into two types (type I and type II), is a rare autosomal recessive lysosomal storage disorder caused by mutations in the *NEU1* gene. Typically, sialidosis type I manifests in late childhood or early adulthood and is characterized by the progressive development of myoclonic ataxia, visual impairment, and the presence of macular cherry-red spots. Conversely, type II presents with more severe symptoms, including developmental delay, hepatosplenomegaly, and dysmorphic facial features, and generally presents at an earlier age [3,4,5]. The considerable range of clinical manifestations, particularly regarding neurological and ophthalmological complications, underscores the complex nature of sialidosis and emphasizes the importance of understanding its genetic foundations [6].

### 1.2. Clinical Presentation and Molecular Pathology

Patients diagnosed with sialidosis frequently present a range of symptoms that significantly impact their overall quality of life. The clinical manifestations of this condition include myoclonus, ataxia, seizures, and visual impairments, with many individuals exhibiting distinctive fundoscopic characteristics, such as macular cherry-red spots, which serve as critical diagnostic markers. Furthermore, abnormalities identified through optical coherence tomography have been observed, underscoring the involvement of both the central nervous system and the retina [7,8,9].

The molecular pathology of sialidosis is significant heterogeneity, with numerous mutations identified within the *NEU1* gene. Over 90 pathogenic variants have been documented, primarily comprising missense mutations compromising the enzyme’s structural integrity and functionality [10,11,12,13,14]. These mutations may result in diminished enzymatic activity, altered subcellular localization, and decreased protein levels, all contributing to the phenotypic variability observed among affected individuals [10,15]. Understanding molecular heterogeneity is fundamental to elucidating the complex pathogenesis of the disease, identifying potential therapeutic targets, and highlighting the importance of genetic analysis for the accurate diagnosis and effective management of sialidosis.

The relationship between mutations in the *NEU1* gene and the resultant clinical features is essential for a comprehensive understanding of sialidosis. Ongoing research into the genetic and molecular mechanisms that underlie this disorder is imperative for enhancing diagnosis, treatment, and patient outcomes. This review aims to elucidate the genetic underpinnings and clinical ramifications associated with sialidosis, highlighting the significance of identifying novel mutations, including compound heterozygous mutations within the *NEU1* gene, as these may offer valuable insights into the disorder’s heterogeneity. By integrating findings from the existing literature alongside a recent case study involving a patient with novel mutations, we endeavor to deepen the understanding of sialidosis, thereby facilitating improved diagnostic and therapeutic approaches for affected individuals.

## 2. Methodology

This study was conducted as a systematic review to analyze the genetic and clinical heterogeneity associated with sialidosis, following the PRISMA guidelines. A comprehensive search was performed across the PubMed, Scopus, and Web of Science databases using the keywords “sialidosis”, “*NEU1* mutations”, “lysosomal storage disorders”, and “gene therapy”. The inclusion criteria encompassed peer-reviewed articles published between 1990 and 2024 that focused on genetic mutations, clinical presentations, and therapeutic strategies related to sialidosis. Case reports, cohort studies, and reviews were included, while studies lacking detailed genetic or clinical data were excluded. Data were synthesized to identify trends and gaps in the literature, emphasizing advancements in diagnostic tools, population-specific variations, and preclinical therapeutic developments. This systematic approach ensured the inclusion of high-quality evidence to enhance the understanding of sialidosis and to inform future research directions.

## 3. Historical Context and Initial Discoveries

Neuraminidase 1 (or sialidase-1) is an enzyme encoded by the *NEU1* gene on chromosome 6p21.33. It has been associated with sialidosis, a rare lysosomal storage condition marked by the buildup of sialic acid caused by insufficient sialidase activity. Initial discoveries in the field identified several variants in the *NEU1* gene that were linked to the wide range of clinical symptoms produced by sialidosis. A study revealed variants in molecular characteristics among *NEU1* mutations, resulting in distinct clinical manifestations. Relevant mutation types that affect the enzymatic activity of the neuraminidase-1 enzyme include missense mutations, exonic duplications, and minor deletions [16].

Type 1 sialidosis, the milder form of the disorder, is predominantly characterized by progressive neurological symptoms. Common manifestations include myoclonus, ataxia, and generalized seizures, often presenting in adolescence or early adulthood. Type II presents in infancy or early childhood with severe systemic involvement, including coarse facial features, skeletal abnormalities, hepatosplenomegaly, and a shorter lifespan than type I patients. Ocular abnormalities, including bilateral macular cherry-red spots, are hallmark diagnostic features. Ophthalmological findings, such as hyper-reflectivity and thickening of the retinal nerve fiber layer on optical coherence tomography (OCT), along with progressive visual impairment, further underscore the multisystemic nature of the disease [17,18,19,20,21,22,23]. Comprehensive cohort studies have facilitated the early recognition of hallmark clinical features, enabling more precise diagnostic strategies [6,11].

## 4. Advances in Genetic Analysis

In recent years, significant advancements in large-scale genome sequencing technologies have revolutionized the detection of pathogenic polymorphisms within the *NEU1* gene, which is fundamental for understanding the genetic basis of sialidosis [24,25,26,27,28,29,30,31,32]. The ability to analyze extensive genomic datasets has empowered researchers to uncover uncommon variants, thereby enhancing our repertoire of known mutations, both novel and previously characterized. These mutations primarily affect the *NEU1* gene, which is crucial for proper sialidase function. For instance, studies have reported a variety of mutations, including missense, nonsense, and splice donor site mutations, that disrupt the enzyme’s function and contribute to diverse clinical phenotypes [11,12,33,34]. The application of recombinant adenoviral expression techniques has allowed researchers to analyze the functional impacts of these mutations on NEU1 enzyme activity and localization [33]. Variant classification based on their structural impacts helps to understand how different mutations influence enzyme functionality and affect clinical outcomes [24]. Further, a study leveraging data from 7595 individuals successfully identified three established mutations alongside three new disease-causing variants [10], demonstrating the efficacy of integrating genomic information with functional evaluations in elucidating the genetic underpinnings of this disorder. Notably, in silico analytical methods have played a pivotal role in characterizing missense and frameshift mutations that compromise sialidase activity. These analyses have not only facilitated the discovery of previously unknown functional alleles but have also underscored the pathogenic implications of such mutations in determining clinical phenotypes in individuals affected by type I sialidosis. The insights gained from these advanced genetic analyses herald a new era for understanding the complexity of sialidosis and the potential for targeted therapeutic strategies. Such novel findings lay the groundwork for future research to elucidate further the genotype–phenotype correlations critical for patient diagnosis, management, and the development of effective interventions.

## 5. Clinical Case Studies and Population-Specific Insights

The clinical manifestations of sialidosis vary depending on a patients’ ethnic origins. Significantly, a comparative investigation has shown notable variations in the occurrence of symptoms and genetic alterations across Caucasian and Asian populations [16]. For example, the analysis of sialidosis patients reveals that although the average age at which myoclonus, ataxia, seizures, and visual symptoms appear are equal in both groups, there are significant differences in terms of cognitive impairment and ocular manifestations. Asian patients exhibit a lower incidence of impaired cognition (21.7%) in comparison to Caucasian patients (50.0%). Additionally, the occurrence of cherry-red patches is also lower in Asians (40.7% compared to 61.1% in Caucasians, P = 0.02) [16].

A comprehensive understanding of the genetic variability and possible population-specific health consequences associated with sialidosis requires a thorough examination of the rates of Neu1 mutation among various ethnic groups. CLIVAR has annotated 94 probable pathogenic variants in the *NEU1* gene (Table S1). Through a comprehensive analysis of the precise sites and types of *NEU1* gene mutations, their corresponding protein alterations, rates among various ethnic groups, and recorded clinical cases, this table facilitates a thorough comprehension of the impact of these variations on drug presentation. It comprises essential clinical information such as the age at which symptoms appear, gender, clinical type, symptoms such as cherry-red patches, and residual neuraminidase activity. Notable findings include missense mutations (e.g., Gly277Arg, Arg294Cys) frequently associated with type I sialidosis, while the frameshift mutations (e.g., S403Tfx*85) are predominantly linked to type II sialidosis. Patients with residual enzymatic activity often present with milder phenotypes. Severe phenotypes correlate with null mutations. An exemplary genetic trait is the existence of a prevalent mutational variant, c.544A > G (p.Ser182Gly), which is primarily observed in Asian patients (particularly the Taiwanese population) but not in Caucasian populations. The observed mutation shows a founder effect within the Taiwanese ethnic group, indicating a significant genetic factor that may impact the disease’s presentation and management [16,35,36].

**Figure 1:**
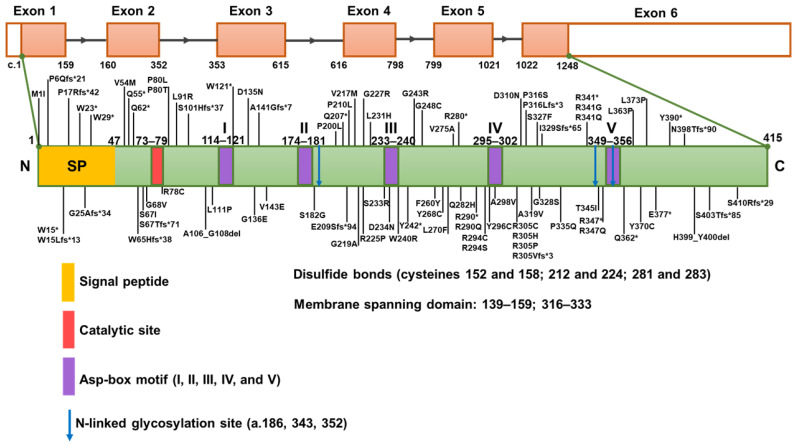
Structural representation and variant locations in the *Neu1* gene. The top part of the figure shows the linear arrangement of exons (orange boxes) and introns (black lines) in the *NEU1* gene. Exons are sequentially numbered from the 5′ to the 3′ end. The bottom part provides critical structural features and functional domains of NEU1 protein. Five conserved Asp-box motifs (I to V) are represented by violet bands. The asterisk (*) denotes a nonsense mutation, resulting in the introduction of a premature stop codon within the gene sequence.

Figure 1 depicts the structural arrangement of the *NEU1* gene, emphasizing the positions of different domains and pathogenic variations. The upper-portion displays the organization of exons, while the below-portion provides a comprehensive description of the protein structure, encompassing the signal peptide (SP) and catalytic domains. Pathogenic variations are identified and shown along the protein sequence, establishing a clear visual link between genetic mutations and their specific locations within the functional domains of NEU1. Changes in the flexibility and local polar contacts of the NEU1 protein can serve as indicators of pathogenicity [24]. The structural changes associated with specific variants, especially whether they maintain some residual enzymatic activities, are crucial in determining the severity of sialidosis. Patients with genetically compound forms (having both mild and severe mutations) tend to exhibit milder forms of sialidosis, supporting the idea that some functional enzyme activity can mitigate severe clinical manifestations [11]. Comprehensive investigations that integrate genetic analysis with functional and structural evaluations are crucial for comprehending the influence of new mutations on the function of the *NEU1* gene and the correlation between genotype and phenotype in sialidosis.

**Figure 2:**
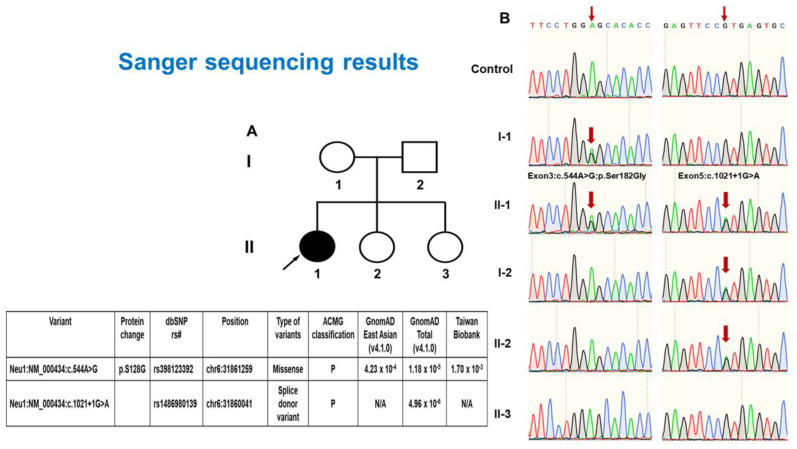
Genetic analysis of the *NEU1* gene in the proband and her family. (**A**) The family pedigree of the proband. The filled black symbol represents the affected proband (II-1), while open symbols represent unaffected family members. The black arrow indicates the proband. (**B**) Sanger sequencing results showing the identified *NEU1* variants. The proband (II-1) carries a compound heterozygosity, including the previously known missense variant exon3:c.544A > G (p.Ser182Gly) inherited from her asymptomatic mother (I-1) and a novel splicing variant exon5:c.1021+1G > A inherited from her asymptomatic father (I-2). The variants are indicated by red arrows. The proband’s younger sister (II-3) also carries the splicing variant exon5:c.1021+1G > A but is unaffected. Sequencing results of the unaffected family members and a control are provided for comparison.

**Figure 3:**
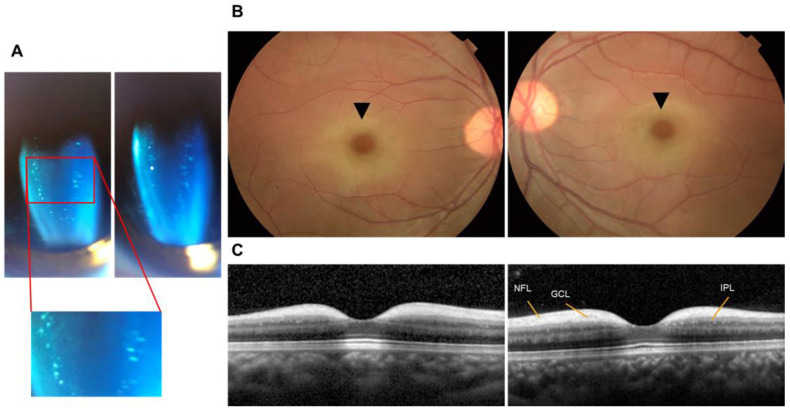
Clinical characteristics of the proband. (**A**) Slit lamp photography showed scattered small punctate opacities in the lens. (**B**) Fundus images revealed cherry-red spots in both maculae (black arrowhead). (**C**) OCT showed hyper-reflectivity and thickening of the nerve fiber layer and ganglion cell layer. NFL: nerve fiber layer. GCL: retinal ganglion cell layer. IPL: inner plexiform layer.

The present investigation involved a patient from Taiwan who exhibited compound heterozygosity for a hitherto undocumented splicing variant c.1021+1G>A and a widely known variant c.544A > G (Figure 2). A female patient, aged 12, appeared with a medical history of visual impairment and amblyopia that began at the age of 4. Further genetic investigation was prompted by observing considerably extended P100 latencies during visual evoked potential (VEP) testing. In the *NEU1* gene, the proband and her asymptomatic mother possessed a harmful missense variant exon3:c.544A > G. In contrast, the proband, her asymptomatic father, and younger sister had a hitherto unreported splicing variant exon5:c.1021+1G > A. Analysis of leukocyte lysosomal enzyme activity showed a notable reduction in neuraminidase-1 activity to 0.021 nmol/mg protein/hour, which is 6.4% of the normal level (reference range 0.326 ± 0.095 nmol/mg protein/hour). This confirms the presence of type 1 sialidosis. Pharmacological treatments provided only partial effectiveness in treating the clinical course characterized by gradual neurological deterioration, myoclonic convulsions, and deteriorating ataxia. An ophthalmological examination revealed punctate lens deposits and bilateral macular cherry-red patches, suggesting the presence of lysosomal storage disease (Figure 3). The patient also presented with myoclonic convulsions, ataxia, and a gradual decline in visual acuity. The diagnosis was validated by a comprehensive examination involving genetic testing, neurophysiological exams, and brain MRI. Neurophysiological studies, including electroencephalogram (EEG) and nerve conduction velocity (NCV) studies, delineated the extent of neurological involvement, revealing focal epileptiform discharges and polyradiculopathy. Brain magnetic resonance imaging (MRI) demonstrated the right parietal lobe with mild surrounding gliosis suggestive of underlying pathological processes. The presence of the compound heterozygous state (exon3:c.544A > G and exon5:c.1021+1G > A) in the patient highlights the intricate genetic components and emphasizes the need for thorough genetic testing in the diagnosis of sialidosis. From a clinical perspective, the case demonstrates the rapid development of vision loss, advancing neurological symptoms, and distinctive eye abnormalities, presenting a more comprehensive description of the disease’s phenotype. The extensive diagnostic paradigm, encompassing genetic analysis, neurophysiological investigations, and imaging, underscores the need for a meticulous assessment in comprehending and controlling sialidosis.

The occurrence of a wide variety of mutations, such as c.880C > A (p.Arg294Ser) [37] and c.679G > A (p.Gly227Arg) [32,35,37,38,39,40,41,42] among various ethnic groups, suggests that genetic drift and selection play a significant role in determining the occurrence of sialidosis and other risk factors associated with the disease. The ethnic disparities have far-reaching consequences for treatment methodologies. Elucidating the genetic foundations of sialidosis in various cultures can result in customized treatment approaches, especially as progress in gene therapy and gene editing persists. For instance, identifying particular mutations within various ethnic groups enables the development of focused treatments that explicitly target the distinct genetic characteristics of these people. Therefore, understanding the phenotypic and genotypic variance among different races is crucial for directing future therapy options for sialidosis and optimizing patient care. Moreover, analyzing the age at which symptoms emerge and the age at which they are diagnosed among different ethnic groups might offer a valuable understanding of the development of sialidosis. It has the potential to guide focused screening and treatment strategies for impacted groups.

## 6. Emerging Therapeutic Strategies for NEU1-Related Diseases

Developing functional NEU1 enzymes for therapeutic applications presents several significant challenges. One primary issue is the effective delivery of the enzyme to the affected tissues. Ensuring that the NEU1 enzyme reaches its target cells and tissues in a functional state is complex due to its specific biological requirements and the diverse nature of tissues affected by *NEU1* mutations. Moreover, NEU1 substrates vary widely among different tissue types, necessitating a highly tailored approach to therapy.

Enzyme replacement therapy (ERT) is a prominent therapeutic strategy being explored for treating *NEU1* mutations. ERT involves administering a recombinant form of the deficient enzyme to restore normal function and alleviate disease symptoms. Research efforts have investigated the impacts of short-term, high-dose ERT in various mouse models, yielding promising results that suggest this approach may not only boost residual NEU1 activity but also mitigate kidney damage associated with the disease [43]. However, one of the primary hurdles is the immunogenicity of the NEU1 enzyme itself, which complicates the application of ERT. The immune response can lead to adverse effects, necessitating a careful approach to treatment design. Researchers must prioritize engineering enzymes with enhanced stability and prolonged half-lives to ensure sustained therapeutic efficacy. Additionally, these formulations should be specifically designed to minimize immune responses, which could otherwise compromise the effectiveness of the therapy.

Small molecule therapies targeting NEU1 have garnered significant attention in recent years, given their potential to treat diseases linked to *NEU1* mutations and deficiencies. These molecules involve diverse biological processes, including cell signaling, adhesion, and immune responses [44]. Recent research has identified several promising small molecules that can stabilize NEU1, enhancing its stability and enzymatic activity. These small molecules bind to specific regions on the NEU1 enzyme, preventing its degradation and maintaining its functional conformation [45]. This stabilization is crucial for ensuring the enzyme’s proper function, especially in pathological conditions where NEU1 activity is compromised. The potential therapeutic applications of NEU1 stimulators are vast, spanning multiple disease categories. For instance, enhancing NEU1 activity could offer new treatment strategies for cardiovascular diseases such as atherosclerosis [46,47,48] and ischemic heart disease [49,50,51], nervous system disorders like Alzheimer’s disease [52] and epileptic seizures, and various cancers including hepatocellular carcinoma and melanoma.

One of the most promising strategies involves applying adeno-associated virus (AAV) vectors as a delivery system for NEU1 and its chaperone, protective protein/cathepsin A (PPCA). In preclinical studies utilizing specific mouse models of sialidosis, the co-administration of two AAV vectors—one containing NEU1 and the other carrying PPCA—demonstrated significant therapeutic outcomes. Treated mice displayed a marked restoration of NEU1 activity across multiple tissues, reduced accumulation of sialylated metabolites, and normalization of lysosomal function [53,54,55]. These encouraging results underscore the potential of AAV-mediated gene therapy to be translated into clinical applications for human patients suffering from sialidosis and related lysosomal storage disorders.

Continued research aims to refine these therapeutic strategies, with key investigations focused on optimizing vector delivery methods and enhancing genetic stability to maximize therapeutic efficacy. Collaborations between researchers, clinicians, and patient advocacy groups are crucial in this endeavor, ensuring that preclinical successes are effectively transitioned into clinical applications. Such cooperative efforts promise to accelerate the development and implementation of these innovative therapies for sialidosis and lay the groundwork for similar strategies in treating other lysosomal storage disorders.

## 7. Limitations and Future Direction

Recent research on NEU1-related diseases, particularly sialidosis, has significantly advanced our understanding of these conditions’ clinical manifestations and genetic variations. However, several limitations in this field must be addressed to enhance future studies. A notable constraint is the often-small sample sizes of patient cohorts, as demonstrated in various studies focusing on specific populations, which may not fully capture the diversity of mutations and clinical presentations across different ethnic groups. Additionally, many studies rely on retrospective analysis, which can introduce biases due to inconsistencies in diagnostic criteria and reporting practices across research publications. Furthermore, the complex interplay of genetic and environmental factors in the disease spectrum is not fully elucidated, limiting the generalizability of findings. To overcome these challenges, future research should prioritize extensive, multicenter collaborative studies that include diverse populations and utilize prospective designs to standardize data collection. Emphasizing genetic testing and cross-disciplinary approaches can also lead to more precise therapeutic strategies and ultimately improve patient outcomes in those affected by NEU1-related diseases.

The other primary concern is the genetic and phenotypic variability among patients, complicating the identification of universal therapeutic approaches. Additionally, many promising therapies, including gene therapy and enzyme replacement strategies, are still in preclinical stages, and their efficacy and safety need rigorous validation through controlled clinical trials. Limited patient populations further constrain the development and testing of these therapies, potentially leading to statistical biases in study outcomes. Future directions should focus on expanding patient registries and fostering multicenter collaborations that allow for comprehensive data collection and analysis. Moreover, employing innovative technologies such as machine learning and bibliometric analysis could streamline drug discovery processes, enabling the identification of new therapeutic candidates tailored to the diverse genetic profiles associated with NEU1-related diseases. There is a vital need for ongoing research into not only therapeutic efficacy but also patient quality of life improvements and integration of supportive care approaches to enhance overall outcomes. 

## 8. Conclusions

The overlapping features between sialidosis and other lysosomal storage disorders complicate early diagnosis, necessitating molecular genetic testing for confirmation. Identifying new mutations in the *NEU1* gene is critical for diagnosing, treating, and investigating sialidosis. This review underscores the importance of genetic analysis in elucidating the complexities surrounding this rare lysosomal storage disorder while emphasizing further exploration into its pathogenic mechanisms to develop tailored therapeutics for affected individuals.

## Data Availability

The original contributions presented in this study are included in the article/Supplementary Materials. Further inquiries can be directed to the corresponding author.

## Supplementary Materials

The following supporting information can be downloaded at: https://www.mdpi.com/article/10.3390/genes16020151/s1, Table S1: Selected set of likely pathogenic and pathogenic variants annotated by CLIVAR in *NEU1* gene.

## References

[1] Bonten E., van der Spoel A., Fornerod M., Grosveld G., d’Azzo A. (1996). Characterization of human lysosomal neuraminidase defines the molecular basis of the metabolic storage disorder sialidosis. Genes Dev..

[2] Pshezhetsky A.V., Richard C., Michaud L., Igdoura S., Wang S., Elsliger M.A., Qu J., Leclerc D., Gravel R., Dallaire L. (1997). Cloning, expression and chromosomal mapping of human lysosomal sialidase and characterization of mutations in sialidosis. Nat. Genet..

[3] Lowden J.A., O’Brien J.S. (1979). Sialidosis: A review of human neuraminidase deficiency. Am. J. Hum. Genet..

[4] Rapin I. (1986). Myoclonus in neuronal storage and Lafora diseases. Adv. Neurol..

[5] Meikle P.J., Hopwood J.J., Clague A.E., Carey W.F. (1999). Prevalence of lysosomal storage disorders. JAMA.

[6] Caciotti A., Melani F., Tonin R., Cellai L., Catarzi S., Procopio E., Chilleri C., Mavridou I., Michelakakis H., Fioravanti A. (2020). Type I sialidosis, a normosomatic lysosomal disease, in the differential diagnosis of late-onset ataxia and myoclonus: An overview. Mol. Genet. Metab..

[7] Kersten H.M., Roxburgh R.H., Danesh-Meyer H.V., Hutchinson D.O. (2016). Optical coherence tomography findings in a patient with type 1 sialidosis. J. Clin. Neurosci..

[8] Michalewska Z., Gajos A., Michalewski J., Nawrocki J., Pshezhetsky A.V., Bogucki A. (2011). Spectral optical coherence tomography in a patient with type I sialidosis. Med. Sci. Monit..

[9] Rosenberg R., Halimi E., Mention-Mulliez K., Cuisset J.M., Holder M., Defoort-Dhellemmes S. (2013). Five year follow-up of two sisters with type II sialidosis: Systemic and ophthalmic findings including OCT analysis. J. Pediatr. Ophthalmol. Strabismus.

[10] Bonardi D., Ravasio V., Borsani G., d’Azzo A., Bresciani R., Monti E., Giacopuzzi E. (2014). In silico identification of new putative pathogenic variants in the NEU1 sialidase gene affecting enzyme function and subcellular localization. PLoS ONE.

[11] Seyrantepe V., Poupetova H., Froissart R., Zabot M.T., Maire I., Pshezhetsky A.V. (2003). Molecular pathology of NEU1 gene in sialidosis. Hum. Mutat..

[12] Penzel R., Uhl J., Kopitz J., Beck M., Otto H.F., Cantz M. (2001). Splice donor site mutation in the lysosomal neuraminidase gene causing exon skipping and complete loss of enzyme activity in a sialidosis patient. FEBS Lett..

[13] de Geest N., Bonten E., Mann L., de Sousa-Hitzler J., Hahn C., d’Azzo A. (2002). Systemic and neurologic abnormalities distinguish the lysosomal disorders sialidosis and galactosialidosis in mice. Hum. Mol. Genet..

[14] Loren D.J., Campos Y., d’Azzo A., Wyble L., Grange D.K., Gilbert-Barness E., White F.V., Hamvas A. (2005). Sialidosis presenting as severe nonimmune fetal hydrops is associated with two novel mutations in lysosomal alpha-neuraminidase. J. Perinatol..

[15] Zhang C., Liao Z., Zhou Y., Su X. (2024). Sialidosis type 1 without cherry-red spots: A case report and literature review. BMJ Neurol. Open.

[16] Fan S.P., Lee N.C., Lin C.H. (2020). Clinical and electrophysiological characteristics of a type 1 sialidosis patient with a novel deletion mutation in NEU1 gene. J. Formos Med. Assoc..

[17] Thomas P.K., Abrams J.D., Swallow D., Stewart G. (1979). Sialidosis type 1: Cherry red spot-myoclonus syndrome with sialidase deficiency and altered electrophoretic mobilities of some enzymes known to be glycoproteins. 1. Clinical findings. J. Neurol. Neurosurg. Psychiatry.

[18] Swallow D.M., Evans L., Stewart G., Thomas P.K., Abrams J.D. (1979). Sialidosis type 1: Cherry red spot-myoclonus syndrome with sialidase deficiency and altered electrophoretic mobility of some enzymes known to be glycoproteins. II. Enzymes studies. Ann. Hum. Genet..

[19] Till J.S., Roach E.S., Burton B.K. (1987). Sialidosis (neuraminidase deficiency) types I and II: Neuro-ophthalmic manifestations. J. Clin. Neuroophthalmol..

[20] Federico A., Battistini S., Ciacci G., de Stefano N., Gatti R., Durand P., Guazzi G.C. (1991). Cherry-red spot myoclonus syndrome (type I sialidosis). Dev. Neurosci..

[21] Palmeri S., Villanova M., Malandrini A., van Diggelen O.P., Huijmans J.G., Ceuterick C., Rufa A., DeFalco D., Ciacci G., Martin J.J. (2000). Type I sialidosis: A clinical, biochemical and neuroradiological study. Eur. Neurol..

[22] Ganguly S., Gabani R.U., Chakraborty S., Ganguly S.B. (2004). Sialidosis type I (cherry red spot-myoclonus syndrome). J. Indian Med. Assoc..

[23] Goldberg M.F. (2008). Macular cherry-red spot and corneal haze in sialidosis (mucolipidosis type 1). Arch. Ophthalmol..

[24] Li Y., Liu Y., Wang R., Ao R., Xiang F., Zhang X., Wang X., Yu S. (2024). Clinical and structural characteristics of NEU1 variants causing sialidosis type 1. J. Mov. Disord..

[25] Pokharel P., Dawadi A., Baral B., Dhungana S., Baskota A., Poudel D.R. (2024). Juvenile sialidosis: A rare case and review of the literature. Ann. Med. Surg..

[26] Flores-Contreras E.A., García-Ortiz J.E., Robles-Espinoza C.D., Zomosa-Signoret V., Becerra-Solano L.E., Vidaltamayo R., Castaneda-García C., Esparza-García E., Molina-Aguilar C., Hernández-Orozco A.A. (2021). Clinical Exome Sequencing Enables Congenital Sialidosis Type II Diagnosis in Two Siblings Presenting with Unreported Clinical Features from a Rare Homozygous Sequence Variant p.(Tyr370Cys) in NEU1. Mol. Syndromol..

[27] Cao L.X., Liu Y., Song Z.J., Zhang B.R., Long W.Y., Zhao G.H. (2021). Compound heterozygous mutations in the neuraminidase 1 gene in type 1 sialidosis: A case report and review of literature. World J. Clin. Cases.

[28] Li X., Zhang Q. (2020). Heterozygous structural variation mimicking homozygous missense mutations in NEU1 associated with presenting clinical signs in eyes alone. Ophthalmic. Genet..

[29] Jiao B., Zhou Z., Hu Z., Du J., Liao X., Luo Y., Wang J., Yan X., Jiang H., Tang B. (2020). Homozygosity mapping and next generation sequencing for the genetic diagnosis of hereditary ataxia and spastic paraplegia in consanguineous families. Parkinsonism. Relat. Disord..

[30] Ahn J.H., Kim A.R., Lee C., Kim N.K.D., Kim N.S., Park W.Y., Kim M., Youn J., Cho J.W., Kim J.S. (2019). Type 1 Sialidosis Patient With a Novel Deletion Mutation in the NEU1 Gene: Case Report and Literature Review. Cerebellum.

[31] Maroofian R., Schuele I., Najafi M., Bakey Z., Rad A., Antony D., Habibi H., Schmidts M. (2018). Parental Whole-Exome Sequencing Enables Sialidosis Type II Diagnosis due to an NEU1 Missense Mutation as an Underlying Cause of Nephrotic Syndrome in the Child. Kidney Int. Rep..

[32] Mohammad A.N., Bruno K.A., Hines S., Atwal P.S. (2018). Type 1 sialidosis presenting with ataxia, seizures and myoclonus with no visual involvement. Mol. Genet. Metab. Rep..

[33] Pattison S., Pankarican M., Rupar C.A., Graham F.L., Igdoura S.A. (2004). Five novel mutations in the lysosomal sialidase gene (NEU1) in type II sialidosis patients and assessment of their impact on enzyme activity and intracellular targeting using adenovirus-mediated expression. Hum. Mutat..

[34] Coutinho M.F., Lacerda L., Macedo-Ribeiro S., Baptista E., Ribeiro H., Prata M.J., Alves S. (2012). Lysosomal multienzymatic complex-related diseases: A genetic study among Portuguese patients. Clin. Genet..

[35] Lukong K.E., Elsliger M.A., Chang Y., Richard C., Thomas G., Carey W., Tylki-Szymanska A., Czartoryska B., Buchholz T., Criado G.R. (2000). Characterization of the sialidase molecular defects in sialidosis patients suggests the structural organization of the lysosomal multienzyme complex. Hum. Mol. Genet..

[36] Lai S.C., Chen R.S., Wu Chou Y.H., Chang H.C., Kao L.Y., Huang Y.Z., Weng Y.H., Chen J.K., Hwu W.L., Lu C.S. (2009). A longitudinal study of Taiwanese sialidosis type 1: An insight into the concept of cherry-red spot myoclonus syndrome. Eur. J. Neurol..

[37] Bonten E.J., Arts W.F., Beck M., Covanis A., Donati M.A., Parini R., Zammarchi E., d’Azzo A. (2000). Novel mutations in lysosomal neuraminidase identify functional domains and determine clinical severity in sialidosis. Hum. Mol. Genet..

[38] Caciotti A., Di Rocco M., Filocamo M., Grossi S., Traverso F., d’Azzo A., Cavicchi C., Messeri A., Guerrini R., Zammarchi E. (2009). Type II sialidosis: Review of the clinical spectrum and identification of a new splicing defect with chitotriosidase assessment in two patients. J. Neurol..

[39] Canafoglia L., Robbiano A., Pareyson D., Panzica F., Nanetti L., Giovagnoli A.R., Venerando A., Gellera C., Franceschetti S., Zara F. (2014). Expanding sialidosis spectrum by genome-wide screening: NEU1 mutations in adult-onset myoclonus. Neurology.

[40] Schene I.F., Kalinina Ayuso V., de Sain-van der Velden M., van Gassen K.L., Cuppen I., van Hasselt P.M., Visser G. (2016). Pitfalls in Diagnosing Neuraminidase Deficiency: Psychosomatics and Normal Sialic Acid Excretion. JIMD Rep..

[41] Arora V., Setia N., Dalal A., Vanaja M.C., Gupta D., Razdan T., Phadke S.R., Saxena R., Rohtagi A., Verma I.C. (2020). Sialidosis type II: Expansion of phenotypic spectrum and identification of a common mutation in seven patients. Mol. Genet. Metab. Rep..

[42] Vanaja M.C., Nurul Jain J.M., Dalal A., Ranganath P. (2023). Long-range PCR amplification-based targeted enrichment & next generation sequencing: A cost-effective testing strategy for lysosomal storage disorders. Indian J. Med. Res..

[43] Wang D., Bonten E.J., Yogalingam G., Mann L., d’Azzo A. (2005). Short-term, high dose enzyme replacement therapy in sialidosis mice. Mol. Genet. Metab..

[44] Du J., Shui H., Chen R., Dong Y., Xiao C., Hu Y., Wong N.K. (2024). Neuraminidase-1 (NEU1): Biological Roles and Therapeutic Relevance in Human Disease. Curr. Issues Mol. Biol..

[45] Bonten E.J., Annunziata I., d’Azzo A. (2014). Lysosomal multienzyme complex: Pros and cons of working together. Cell Mol. Life Sci..

[46] Sieve I., Ricke-Hoch M., Kasten M., Battmer K., Stapel B., Falk C.S., Leisegang M.S., Haverich A., Scherr M., Hilfiker-Kleiner D. (2018). A positive feedback loop between IL-1β, LPS and NEU1 may promote atherosclerosis by enhancing a pro-inflammatory state in monocytes and macrophages. Vascul. Pharmacol..

[47] Demina E.P., Smutova V., Pan X., Fougerat A., Guo T., Zou C., Chakraberty R., Snarr B.D., Shiao T.C., Roy R. (2021). Neuraminidases 1 and 3 Trigger Atherosclerosis by Desialylating Low-Density Lipoproteins and Increasing Their Uptake by Macrophages. J. Am. Heart Assoc..

[48] White E.J., Gyulay G., Lhoták Š., Szewczyk M.M., Chong T., Fuller M.T., Dadoo O., Fox-Robichaud A.E., Austin R.C., Trigatti B.L. (2018). Sialidase down-regulation reduces non-HDL cholesterol, inhibits leukocyte transmigration, and attenuates atherosclerosis in ApoE knockout mice. J. Biol. Chem..

[49] Guo Z., Fan D., Liu F.Y., Ma S.Q., An P., Yang D., Wang M.Y., Yang Z., Tang Q.Z. (2022). NEU1 Regulates Mitochondrial Energy Metabolism and Oxidative Stress Post-myocardial Infarction in Mice via the SIRT1/PGC-1 Alpha Axis. Front. Cardiovasc. Med..

[50] Chen Q.Q., Ma G., Liu J.F., Cai Y.Y., Zhang J.Y., Wei T.T., Pan A., Jiang S., Xiao Y., Xiao P. (2021). Neuraminidase 1 is a driver of experimental cardiac hypertrophy. Eur. Heart J..

[51] Heimerl M., Sieve I., Ricke-Hoch M., Erschow S., Battmer K., Scherr M., Hilfiker-Kleiner D. (2020). Neuraminidase-1 promotes heart failure after ischemia/reperfusion injury by affecting cardiomyocytes and invading monocytes/macrophages. Basic Res. Cardiol..

[52] Fremuth L.E., Hu H., van de Vlekkert D., Annunziata I., Weesner J.A., d’Azzo A. (2025). Neuraminidase 1 regulates neuropathogenesis by governing the cellular state of microglia via modulation of Trem2 sialylation. Cell Rep..

[53] Bonten E.J., Yogalingam G., Hu H., Gomero E., van de Vlekkert D., d’Azzo A. (2013). Chaperone-mediated gene therapy with recombinant AAV-PPCA in a new mouse model of type I sialidosis. Biochim. Biophys. Acta.

[54] van de Vlekkert D., Hu H., Weesner J.A., Fremuth L.E., Brown S.A., Lu M., Gomero E., Campos Y., Sheppard H., d’Azzo A. (2024). AAV-mediated gene therapy for sialidosis. Mol. Ther..

[55] Hwu W.L., Chang K., Liu Y.H., Wang H.C., Lee N.C., Chien Y.H. (2024). Gene therapy corrects the neurological deficits of mice with sialidosis. Gene Ther..

